# Recombinant Expression and Antimicrobial Mechanism of Cysteine-Rich Antimicrobial Peptides from *Tigriopus japonicus* Genome

**DOI:** 10.3390/md24010045

**Published:** 2026-01-16

**Authors:** Dan Pu, Hongwei Tao, Jingwei Pang, Huishao Shi, Junjian Wang, Wei Zhang

**Affiliations:** College of Marine Science, Beibu Gulf University, Qinzhou 535011, China; p15882749472@163.com (D.P.); taohongweibbgu@126.com (H.T.); 18977728207@163.com (J.P.);

**Keywords:** antimicrobial peptides, cysteine-rich, antibacterial mechanism, *Tigriopus japonicus*

## Abstract

The misuse of antibacterial agents has contributed to the growing prevalence of antibiotic resistance, highlighting an urgent need to explore alternative anti-infection therapeutic strategies. Antimicrobial peptides (AMPs) are naturally occurring molecules. They exhibit broad-spectrum antimicrobial activity and represent promising candidates for the development of novel therapeutics. A cysteine-rich antimicrobial peptide was identified and characterized from the genome of *Tigriopus japonicus* and designated “*Tj*Rcys1”. The precursor form of *Tj*Rcys1 comprises 96 amino acids. Structural analyses of *Tj*Rcys1 revealed random coils, two α-helices, and two β-strands. Recombinant *Tj*Rcys1 had inhibitory effects upon *Staphylococcus aureus* and *Bacillus* sp. T2, with a minimum inhibitory concentration of 64 μM for both. *Tj*Rcys1 did not show complete inhibition against *Vibrio alginolyticus*, *Klebsiella pneumoniae*, or *Aeromonas hydrophila* at 64 μM, but it did slow their growth rate. *Tj*Rcys1 could disrupt the permeability of the cell membrane of *S. aureus*. Transcriptomic analyses indicated that *Tj*Rcys1 could interfere with the ribosome biosynthesis and nucleotide metabolism of *K. pneumoniae*. Our results provide a valuable reference for the development of new AMPs and optimization of their design.

## 1. Introduction

In recent years, the overuse of antibiotics has led to increasingly severe issues, such as bacterial resistance and drug residues [1,2]. These problems have resulted in significant economic losses and exacerbated farming burdens, but also posed potential threats to ecological environments [3,4]. Consequently, reducing antibiotic usage has become imperative.

Antimicrobial peptides (AMPs) are up-and-coming alternatives to conventional antibiotic drugs [5]. AMPs serve as essential elements of the innate immune system, providing primary protection against microbial pathogens such as bacteria and fungi [6,7]. Arthropods account for >50% of all documented AMPs, but crustacean-derived AMPs remain relatively understudied [6,7,8]. The APD3 database currently lists only 76 crustacean AMPs since the first identification in *Carcinus maenas* in 1996 [9,10]. Considering the extensive species diversity within crustaceans, this taxonomic group represents a promising (yet underexplored) resource for the discovery of novel AMPs.

Cysteine-rich AMPs represent one of the most evolutionarily conserved and extensively distributed classes of peptide molecules. They are characterized by significant structural variation and a wide range of antimicrobial properties [11,12,13]. Prominent members of this family encompass defensins, charybdotoxins, tachyplesins, and crustins [12,14,15]. These peptides typically exert their effects by compromising the integrity of microbial cytoplasmic membranes or by entering microbial cells and targeting essential intracellular processes, including protein synthesis and DNA replication [16,17].

*Tigriopus* species (Crustacea) inhabit microbially diverse environments such as freshwater ecosystems, marine waters, and estuarine sediments rich in organic humus [18]. These microcrustaceans face continuous exposure to microorganisms in their natural habitats [19]. Invertebrates lack adaptive immunity, so *Tigriopus* species rely exclusively on innate immune mechanisms for pathogen defense [6]. This evolutionary pressure suggests they may have developed potent AMPs as crucial components of their immune repertoire.

We identified *Tj*Rcys1, a novel cysteine-dense AMP from *Tigriopus japonicus*. To evaluate its potential application, we expressed recombinant *Tj*Rcys1 and assessed its antibacterial activity and mechanism of action using molecular experiments and transcriptomic analyses.

## 2. Results

### 2.1. Screening of AMPs with High Cysteine Content

Based on our research methods described previously [20], we used constructed pipeline to search for cysteine-rich AMPs in the genomic files of *T. japonicus*. The protein annotation of the *T. japonicus* genome (GCA_010645155.1) identified 25,143 predicted protein sequences [18]. In general, AMPs consist of ~100 amino acid residues [21]. Of the annotated protein sequences, 2296 were found to be shorter than 100 amino acids. Among sequences of <100 amino acids, only four sequences had a cysteine content >10% (Supplementary Table S1). Cysteine-rich AMPs are typically classified as “cationic AMPs”. Among them, only one peptide was predicted to be a cationic AMP by an AMP calculator and predictor of the APD3 database. Thus, this sequence was named “*Tj*Rcys1”.

### 2.2. Sequence and Structural Characterization of TjRcys1

**Figure 1:**
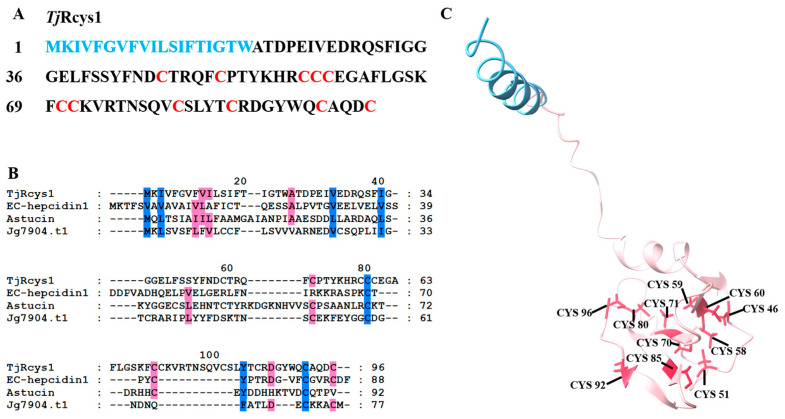
Sequence and structural analyses of *Tj*Rcys1. (**A**) Amino-acid sequence of *Tj*Rcys1. The signal-peptide region is shown in blue, and cysteine residues are labeled in red. (**B**) Alignment results between *Tj*Rcys1 and the three sequences most similar to it in the APD3 database. the residues that are ≥75% identical among the aligned sequences are shaded blue, the residues that are ≥50% identical among the aligned sequences are shaded pink. (**C**) Predicted three-dimensional structure of *Tj*Rcys1 using AlphaFold2 (https://colab.research.google.com/github/sokrypton/ColabFold/blob/main/AlphaFold2.ipynb; accessed on 1 September 2025). Cysteine residues are highlighted in red, and the signal-peptide region is shown in blue.

*Tj*Rcys1 consists of 96 amino acids (Figure 1A). The 19 amino acids at its N-terminal represent the signal-peptide region (Figure 1A). Analyses of sequence alignment using the BLASTP (v 2.15.0) program in the APD3 database (accessed 7 November 2025) revealed that the mature peptide of *Tj*Rcys1 is similar to EC-hepcidin1, with a similarity percentage of 31.82% (Figure 1B). *Tj*Rcys1 was modeled using Alphafold2.3, and it consisted of two α-helices, two β-sheets, and some coils (Figure 1C). The analysis identified a sequence segment (CCCEGAFLGSKFCCKV) that displays features characteristic of a γ-core motif. The molecular weight of *Tj*Rcys1 was ~10.93 kDa, and the protein isoelectric point was 6.51, as determined by ProtParam (https://web.expasy.org/protparam/, accessed on 7 November 2025). The net charge of *Tj*Rcys1 was +0.25. The grand average hydropathy (GRAVY) was 0.06. The protein-binding potential (Boman Index) was 1.26 kcal/mol. *Tj*Rcys1 contained 11 cysteine residues (Figure 1B,C).

### 2.3. MD Simulations Results

Binding to the surface of bacteria is the prerequisite for AMPs to exert their effects. Our previous research using MD simulations [20,22,23] has shown that the cysteine-rich AMPs *Pp*Rcys1, *Pp*Crus-SWD1, and *Aa*Crus1 can approach the cell membrane actively and embed within it [20,22,23]. Based on that discovery, to further predict whether *Tj*Rcys1 also has antibacterial functions, we adopted MD simulations for research.

**Figure 2:**
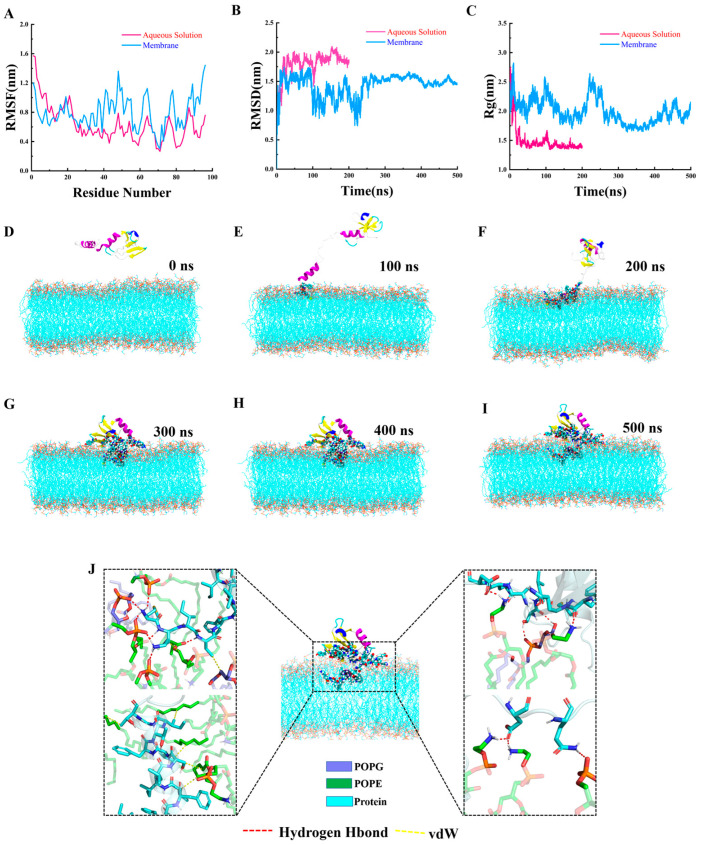
Molecular dynamics (MD) simulations. (**A**) Root mean square fluctuations (RMSF) of *Tj*Rcys1 in aqueous solutions and on membranes. (**B**) The root mean square distance (RMSD) of *Tj*Rcys1 in aqueous solution and on membranes. (**C**) Radius of gyration (Rg) of *Tj*Rcys1 in aqueous solutions and membranes. (**D**–**I**) Dynamic membrane binding of *Tj*Rcys1 during MD simulations (0–500 ns). (**J**) Hydrogen bonds and van der Waals interactions between *Tj*Rcys1 and membranes at 500 ns.

The root mean square distance (RMSD) and root mean square fluctuation (RMSF) are widely used metrics for characterizing the structural dynamics of proteins. The RMSD provides a measure of the overall structural stability of the peptide during the simulation relative to its starting conformation. A stable or converging RMSD profile suggests that the peptide has reached a relatively steady state in the membrane environment, which is crucial for interpreting subsequent functional interactions [24,25]. The RMSF, on the other hand, quantifies the flexibility of individual residues. In the context of AMP–membrane interactions, residues exhibiting high flexibility often correspond to key functional regions, such as those that adapt to the lipid bilayer interface, participate in membrane penetration, or facilitate peptide reorientation [25,26]. Figure 2 presents the RMSF profiles of amino acid residues in AMPs, as well as the RMSD trajectories of these peptides in two distinct systems throughout the MD simulation. As shown in Figure 2A, the RMSF values of most amino acid residues in aqueous solution (pink line) were lower than those observed on the bacterial membrane (blue line), indicating reduced flexibility of the peptide in solution. The membrane of the bacteria consists of 1-palmitoyl-2-oleoyl-sn-glycero-3-phosphoethanolamine (POPE) and 1-palmitoyl-2-oleoyl-sn-glycero-3-phosphoglycerol (POPG). This phenomenon could be attributed to a gradual conformational compaction of the AMP, wherein specific residues become “buried” within the core structure, thereby restricting their mobility. In contrast, when the peptide was associated with the cell membrane, specific residues engaged in dynamic interactions with the membrane surface, migrating laterally and exploring favorable binding sites, resulting in increased residue flexibility. Notably, residues located between positions 0 and 10 exhibited relatively low RMSF values, suggesting limited mobility due to their anchoring role within the hydrophobic core of the membrane. These residues contributed to the stable integration of the membrane. Conversely, residues interacting with the membrane surface displayed higher flexibility. Figure 2B illustrates that the RMSD value of the AMP in aqueous solution was initially higher (pink line), reflecting the substantial conformational rearrangements required to attain a thermodynamically stable state. Following equilibration, the RMSD stabilized at ~1.80 nm. In comparison, the RMSD on the cell membrane (blue line) was consistently lower, indicating that structural deviations from the initial conformation were minor upon membrane adsorption. However, achieving the optimal binding conformation on the membrane required a longer timescale, with equilibrium being reached after ~300 ns. After this point, the RMSD converged to a stable value of ~1.45 nm, reflecting a more rigid and well-defined structural arrangement.

The radius of gyration (Rg) serves as an indicator of changes in the “structural compactness” of proteins during kinetic processes. As shown in Figure 2C, the Rg of *Tj*Rcys1 in aqueous solution (red line) was lower, stabilizing at ~1.40 nm after equilibrium. In contrast, the Rg of *Tj*Rcys1 on the cell membrane (blue line) stabilized at ~1.90 nm upon reaching equilibrium. This difference indicates that the peptide structure was more compact in aqueous solution than on the cell membrane. The AMPs retained a certain degree of compactness when associated with the membrane, but their conformation appeared comparatively less compact in this environment. This behavior can be attributed to the inward aggregation of *Tj*Rcys1 in aqueous solution. However, on the cell membrane, interactions between specific amino acid residues and the membrane surface restrict the complete inward collapse, resulting in a more extended conformation.

Figure 2D–I present a representative “snapshot” from the 500 ns MD simulation of *Tj*Rcys1 interacting with the cell membrane. Initially, *Tj*Rcys1 underwent random diffusion in the aqueous phase. Upon contact between specific amino acid residues of the AMPs and the cell membrane, these residues became stably anchored to the membrane surface through strong interactions. Driven by these interactions, the entire AMP was drawn progressively towards the membrane, ultimately resulting in complete adsorption onto its surface. Furthermore, specific amino acid residues penetrated the hydrophobic core of the membrane, establishing interactions with lipid tails. By the end of the 500 ns simulation, *Tj*Rcys1 had achieved stable binding to the cell membrane, as evidenced in Figure 2I,J.

### 2.4. Recombinant Expression and Purification of TjRcys1

**Figure 3:**
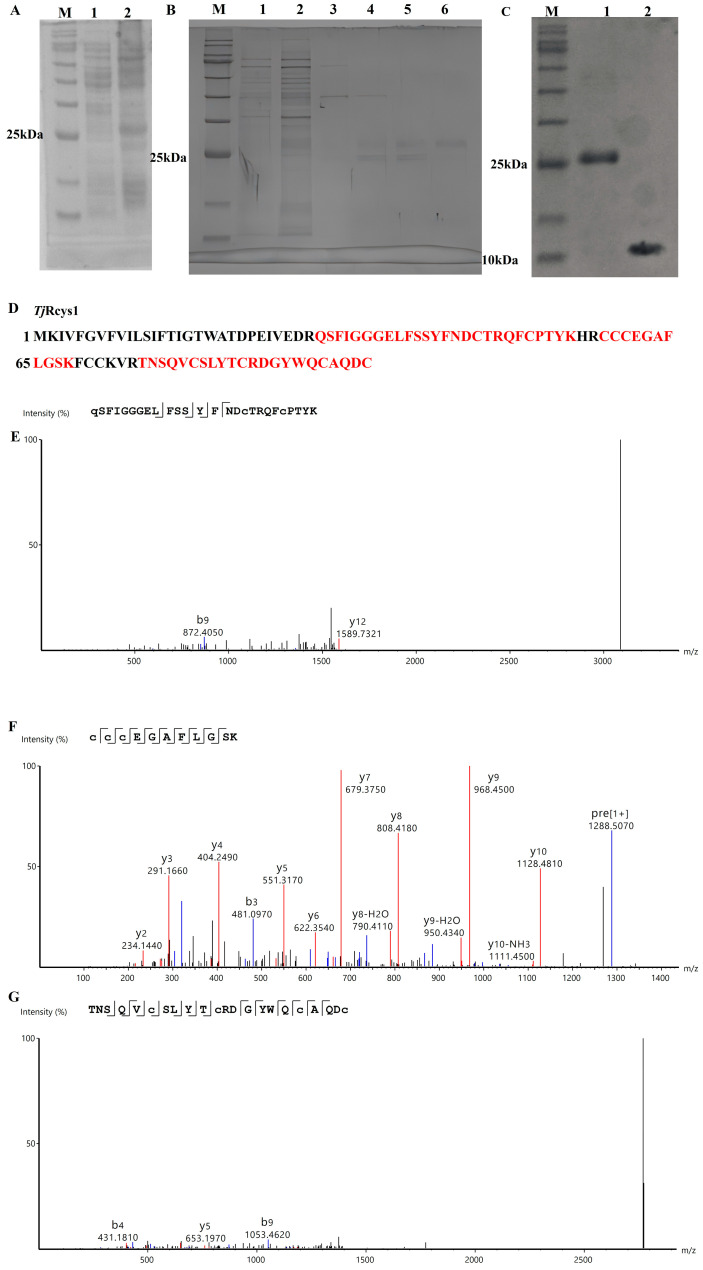
Acquisition and mass spectrometry (MS) of r*Tj*Rcys1. (**A**) SDS–PAGE of recombinant *Tj*Rcys1 (r*Tj*Rcys1) fused with a His-SUMO tag in *Escherichia coli*. Lane M, protein marker; lane 1, total protein obtained from *E. coli* without IPTG induction; lane 2, total protein obtained from *E. coli* with IPTG induction. (**B**) His-SUMO-*Tj*Rcys1 was purified via nickel column chromatography. Lane M, protein marker; lane 1, protein not caught by the nickel column; lane 2, equilibration buffer; lane 3, eluent with 100 mM of imidazole; lane 4, eluent with 200 mM of imidazole; lane 5, eluent with 300 mM of imidazole; lane 6, eluent with 500 mM of imidazole. (**C**) SDS–PAGE of r*Tj*Rcys1 without the SUMO tag. Lane M, protein marker; lane 1, His-SUMO-*Tj*Rcys1 before treatment with SUMO enzyme; lane 2, tag-free r*Tj*Rcys1. (**D**) Alignment of MS results with the r*Tj*Rcys1 sequence. The red area compares MS results with the r*Tj*Rcys1 sequence. (**E**–**G**) MS of “QSFIGGGELFSSYFNDCTRQFCPTYK”, “CCCEGAFLGSK”, and “TNSQVCSLYTCRDGYWQCAQDC”, respectively. The red, blue, and black lines are the y ions, b ions, and noise signals detected by MS, respectively.

The SUMO tag serves to mitigate the cytotoxic effects of target proteins on host cells, reducing the formation of inclusion bodies, while also being efficiently cleaved off by SUMO-specific proteases [27,28]. Sodium dodecyl sulfate–polyacrylamide gel electrophoresis (SDS–PAGE) demonstrated distinct protein expression profiles in bacterial lysates. SDS–PAGE revealed prominent bands exceeding 25 kDa before and after induction with isopropyl-β-D-thiogalactoside (IPTG) (Figure 2A). This observed band aligned with the predicted molecular weight of the His-SUMO-*Tj*Rcys1 fusion construct, comprising the His-SUMO moiety (~18 kDa) and *Tj*Rcys1 (10.93 kDa). The purified His-SUMO-*Tj*Rcys1 protein was eluted from a nickel-affinity column using imidazole-based gradient elution, with optimal elution achieved at 500 μM of imidazole (Figure 3B, lane 6). Following enzymatic cleavage mediated by SUMO protease, the recombinant *Tj*Rcys1 (r*Tj*Rcys1), devoid of the His-SUMO tag, was generated and confirmed by SDS–PAGE to migrate at a size >10 kDa (Figure 3C).

### 2.5. Identification of rTjRcys1 by Liquid Chromatography–Mass Spectrometry (LC-MS)

The sequence of r*Tj*Rcys1 was analyzed using Mascot Distiller (v2.7) [29]. Three distinct peptides were identified with an amino acid sequence coverage of 61.46% (Figure 3D–G). These results confirmed that the fusion protein was expressed in the prokaryotic system, and that the His-SUMO tag was cleaved by SUMO protease, yielding recombinant r*Tj*Rcys1 free of exogenous amino acid residues.

### 2.6. Antimicrobial Activity of rTjRcys1

**Figure 4:**
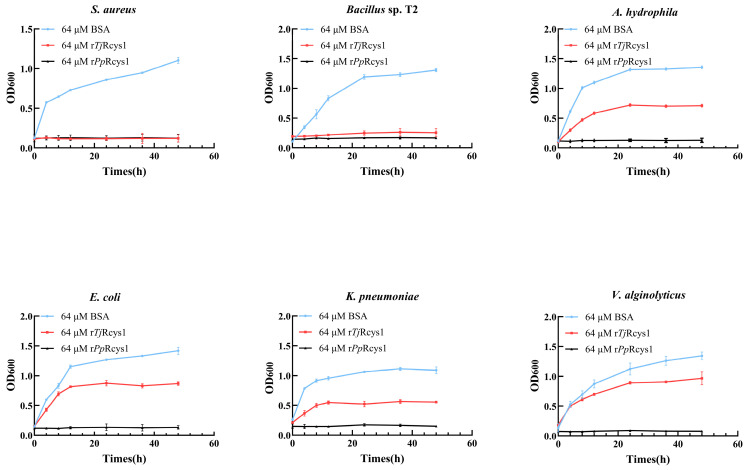
Influence of r*Tj*Rcys1 on the growth rate of *S. aureus*, *Bacillus* sp. T2, *A. hydrophila, K. pneumoniae, E. coli,* and *V. alginolyticys*. Bacterial growth curves were generated by measuring OD_600_ at 0, 4, 8, 12, 24, and 48 h in the presence of 64 μM of r*Tj*Rcys1. The experiment was conducted with three biological replicates, each consisting of three technical replicates. Bovine serum albumin (64 μM) and r*Pp*Rcys1 (64 μM) were used as controls.

Recombinant *Tj*Rcys1 could inhibit the growth of Gram-positive bacteria such as *Staphylococcus aureus* and *Bacillus* sp. T2 at a concentration of 64 μM (Table 1). However, at the same concentration, this r*Tj*Rcys1 did not show complete inhibition against Gram-negative bacteria (*Aeromonas hydrophila*, *Klebsiella pneumoniae*, *E. coli*, and *Vibrio alginolyticus*), but it did slow their growth rate (Figure 4). *Pp*Rcys1 is a cysteine-rich antimicrobial peptide identified initially by our research team in *Pollicipes pollicipes.* Recombinant *Pp*Rcys1 (r*Pp*Rcys1) demonstrates broad-spectrum antibacterial activity against both Gram-positive and Gram-negative bacterial strains [20]. Accordingly, r*Pp*Rcys1 was selected as the control agent in this study.

### 2.7. Membrane Mimetic-Binding Activity of rTjRcys1 and Its Effect on the Permeability of Bacterial Membranes

**Figure 5:**
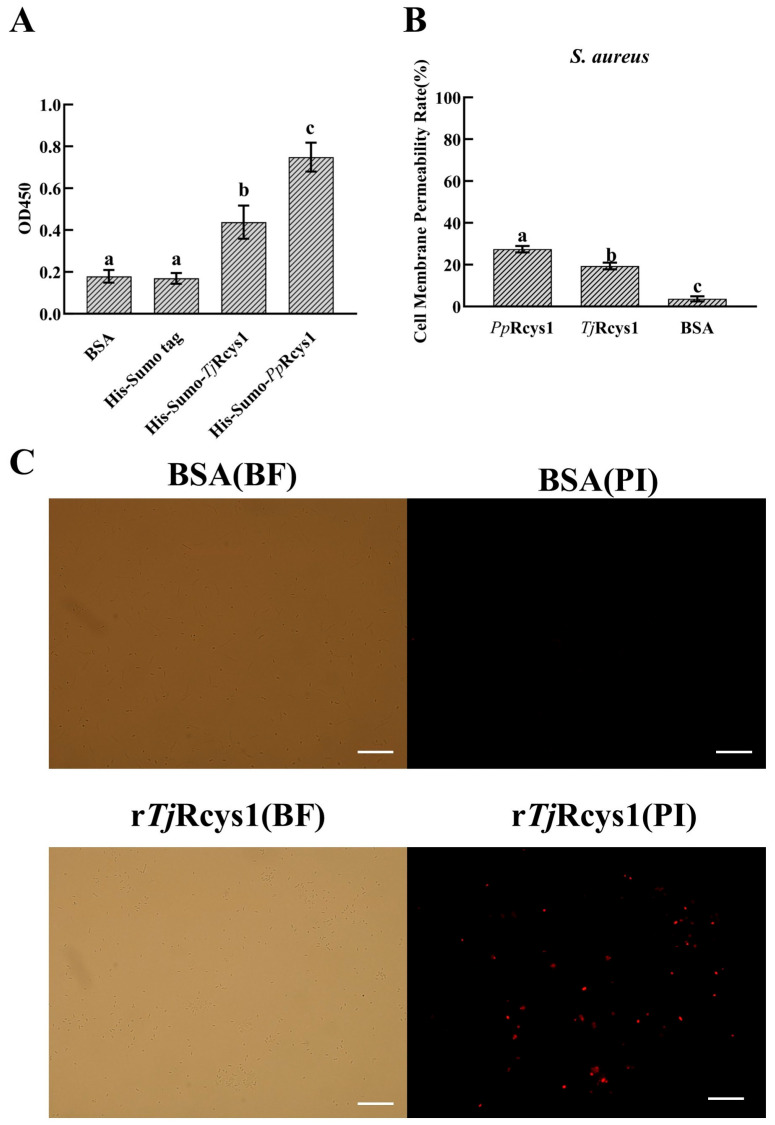
Membrane mimetic-binding activity of r*Tj*Rcys1 and its effect upon the permeability of bacterial membranes. (**A**) Assay to measure membrane mimetic-binding activity. His-Sumo-*Pp*Rcys1 served as the positive control. The His-SUMO tag served as the negative control. (**B**) Impact of r *Tj*Rcys1 on the membrane permeability of *S. aureus*. Recombinant *Pp*Rcys1 (r*Pp*Rcys1) served as the control. Bovine serum albumin acted as the negative control. Experiments were conducted using three biological replicates, each with three technical replicates. Distinct lowercase letters (a, b, c) denote significant differences at *p* < 0.05, while groups labeled with the same letter do not show a significant difference (*p* > 0.05). (**C**) The effect of r*Tj*Rcys1 on bacterial cell membrane integrity. About 1 × 10^6^ CFU·mL^−1^ bacteria were incubated with MIC of r*Tj*Rcys1 for 4 h. PI: the cells were stained with PI and observed for PI uptake with a fluorescence microscope; BF: the bright field image. The scales are 50 μm (White line).

His-SUMO-*Tj*Rcys1, containing the His-SUMO tag, was used for the microorganism-binding assay. The negative control His-SUMO tag was unable to bind to membrane mimics (POPE: POPG) at a 3:1 ratio. r*Tj*Rcys1 could combine with membrane mimics compared with r*Pp*Rcys1, and the binding activity of r*Tj*Rcys1 with membrane mimics decreased by 41.28% (Figure 5A).

Upon disruption of the integrity of microbial membranes, intracellular lactate dehydrogenase (LDH) is released into the extracellular environment, serving as a marker for increased permeability of the bacterial membrane. After 2 h of treatment with r*Tj*Rcys1 at its MIC, the membrane permeability of *S. aureus* was determined to be 19.36%. It had decreased by 29.40% compared with the effect elicited by r*Pp*Rcys1 (Figure 5B).

Propidium iodide (PI) cannot cross the membranes of bacteria that maintain intact cellular envelopes, but it selectively permeates those with damaged membrane structures and then intercalates into intracellular DNA. The results of PI staining revealed significant uptake of the dye in *S*. *aureus* following treatment with r*Tj*Rcys1 (Figure 5C), indicating that r*Tj*Rcys1 induces disruption of bacterial membrane integrity.

### 2.8. Effects of rTjRcys1 upon Bacterial Morphology

**Figure 6:**
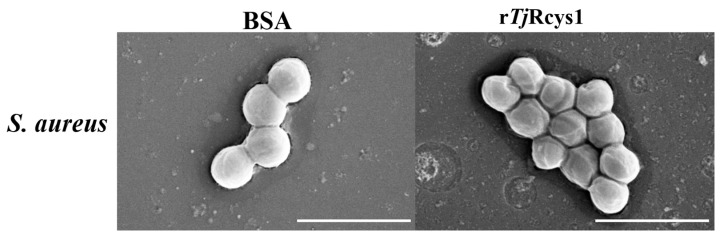
Morphological alterations in bacterial cells following treatment with r*Tj*Rcys1. Bacteria at ~10^6^ CFU/mL were exposed to the MIC of r*Tj*Rcys1 for 2 h and examined using scanning electron microscopy. Bovine serum albumin served as the control. The scales are 2 μm (White line).

Scanning electron microscopy (SEM) revealed that r*Tj*Rcys1 induced significant morphological alterations in bacterial cells compared with those in the untreated control group. *S. aureus* exhibited abnormally irregular cellular morphology (Figure 6).

### 2.9. Effect of rTjRcys1 upon the Activity of K. pneumoniae Cells

**Figure 7:**
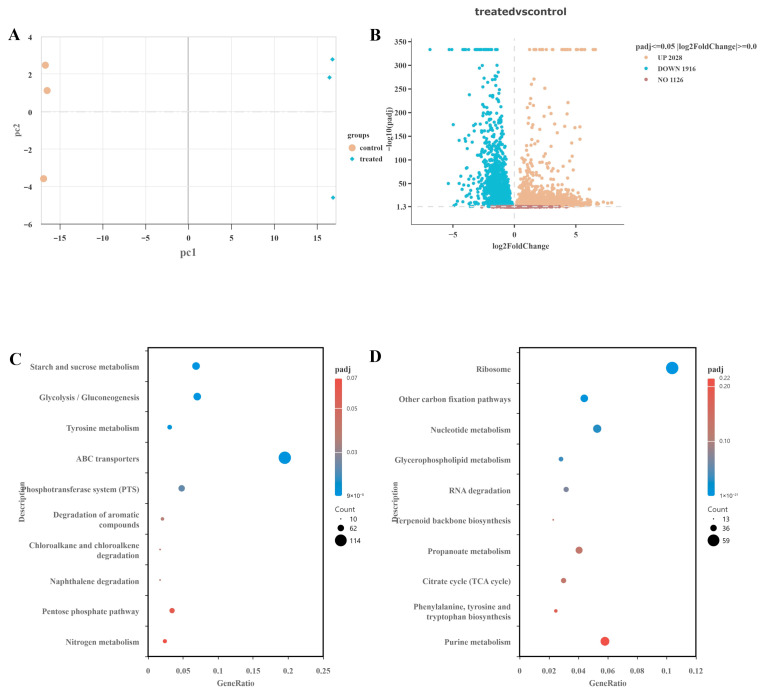
Transcriptome analysis results. (**A**) PCA result. (**B**) DEGs between r*Tj*Rcys1-treated and untreated control groups. (**C**) KEGG enrichment of significantly up-regulated genes. (**D**) KEGG enrichment of significantly down-regulated genes.

It was observed that r*Tj*Rcys1 can reduce the growth rate of Gram-negative bacteria, although it does not fully inhibit bacterial proliferation. This finding suggests that the mechanism of action of r*Tj*Rcys1 may extend beyond simple physical disruption of the cell membrane and likely involves complex intracellular interactions. Therefore, *K. pneumoniae* was selected for the gene expression profiling study. The impact of r*Tj*Rcys1 treatment on the gene-expression profile of *K. pneumoniae* was evaluated through transcriptome sequencing in comparison with the control group. Principal component analysis (PCA, Figure 7A) revealed a clear separation in global patterns of gene expression between the treatment group and control group, indicating substantial transcriptomic alterations induced by treatment. Differential expression analysis (Figure 7B) identified 3944 differentially expressed genes (DEGs): 2028 with upregulated expression and 1916 with downregulated expression.

Analyses of functional enrichment were conducted to explore the potential biological implications of these DEGs. Genes with upregulated expression (Figure 7C) were significantly enriched in metabolic pathways such as “starch and sucrose metabolism” and “glycolysis/gluconeogenesis”, suggesting that treatment may enhance carbohydrate synthesis and degradation processes. Genes with downregulated expression (Figure 7D) were predominantly associated with pathways such as “ribosome”, “nucleotide metabolism”, and “glycerophospholipid metabolism”, suggesting potential inhibitory effects on protein synthesis, nucleic acid metabolism, and specific lipid metabolism-related functions.

## 3. Discussion

AMPs are integral components of innate immune defenses, exhibiting broad-spectrum activity against bacteria, fungi, and viruses. AMPs are promising alternatives to conventional antimicrobial agents [30,31,32]. Due to the vast biodiversity and distinctive ecological niches of marine species, AMPs isolated from marine sources have attracted significant attention as valuable leads in the development of new bioactive compounds [5,6].

A novel cysteine-rich antimicrobial peptide, *Tj*Rcys1, was screened and identified from the genome of *T. japonicus*. MD simulations demonstrated that *Tj*Rcys1 interacted with the bacterial cell membrane via specific amino acid residues and, ultimately, achieved stable anchoring at the membrane surface, a process potentially integral to its antibacterial mechanism. Recombinant *Tj*Rcys1 was expressed efficiently and purified using a prokaryotic expression system, providing a solid foundation for subsequent functional characterization.

Previously, we developed a preliminary screening script suitable for cysteine-rich AMPs (compatible with the Windows system) [20], and used it to obtain the candidate peptide: *Tj*Rys1. However, the net charge of *Tj*Rcys1 is only +0.25. Compared with typical AMPs such as *Pp*Rcys1, *Aa*Crus1, and cecropins, which carry multiple positive charges [20,22], whether it truly possesses antibacterial activity merits in-depth verification. MD simulation, as a powerful computational tool, has demonstrated significant value in the efficient screening of AMPs [25,26]. This method can simulate the dynamic interaction between AMPs and cell membranes at the atomic scale, revealing their possible mechanism of action (e.g., transmembrane perforation and disruption of membrane stability) among other key processes [26,33]. By analyzing parameters such as conformational changes, binding free energy, and lipid order degree in the simulated trajectory, researchers can quantitatively evaluate the efficiency of membrane targeting, selectivity, and potential toxicity of different AMPs. This strategy enables virtual screening and rational design of many candidate sequences before experimentation [24,34]. Crot-1 is a highly efficient AMP screened out by combining artificial intelligence with MD simulation [35]. Based on this premise, we adopted MD simulations to calculate and evaluate the antibacterial potential of *Tj*Rcys1.

The first 19 amino acids of *Tj*Rcys1 were signal peptides, followed by mature peptides. When organisms secrete exocrine proteins, they usually remove signal peptides [36]. However, the mature peptide of *Tj*Rcys1 had a net charge of −0.75. It was predicted to be an AMP in the APD3 database, but this charge property remains puzzling. To determine whether the signal peptide should be retained during heterologous expression, we conducted MD simulations. The latter showed that the signal peptide region of *Tj*Rcys1 first came into contact with the membrane and had entered the membrane entirely by 500 ns. In contrast, the vast majority of mature peptides remained outside the membrane simultaneously. Therefore, we retained the signal-peptide portion in the heterologous expression. During heterologous expression, we selected a larger His-SUMO tag. The latter can protect steric hindrance for the potentially unstable N-terminal region to maintain its native N-terminal structure, and may prevent the recognition and cleavage of this region by signal peptidases [37,38]. Studies have shown that specific signal peptides can be used as targets of drug design [39]. Whether the signal peptide of *Tj*Rcys1 alone has antibacterial activity merits further exploration. The present study employs a recombinant *Tj*Rcys1 protein that encompasses the N-terminal signal peptide for initial functional characterization. However, a direct experimental comparison between the biological activity of this full-length construct and that of the predicted mature peptide has not yet been conducted. Such a comparative analysis will be critical in future investigations to conclusively identify the biologically active form of *Tj*Rcys1 under physiological conditions.

The experiment on antibacterial activity indicated that *Tj*Rcys1 had a significant inhibitory effect upon Gram-positive bacteria (MIC = 64 μM) but did not show complete inhibition on the tested Gram-negative bacteria, only delaying their growth rate. MD simulations demonstrated that *Tj*Rcys1 could bind to the components of bacterial cell membranes. The experiment on membrane-binding activity verified the conclusions from MD simulations, supporting the speculation that *Tj*Rcys1 may cause bacterial death by disrupting their cell membrane barriers. Membrane-permeation experiments and SEM confirmed that *Tj*Rcys1 could cause cell membrane damage in *S. aureus*. The cell wall of Gram-positive bacteria is composed of components such as peptidoglycan and teichoic acid. The latter is conducive to the binding and destruction of the cell wall by cationic AMPs through electrostatic attraction [40,41]. Gram-negative bacteria have a bilayer membrane structure. Lipopolysaccharide on the outer layer forms a barrier, which can prevent most AMPs from entering the cell interior. In addition, the peptidoglycan layer of Gram-negative bacteria is relatively thin, and a periplasmic space is formed between the outer membrane and peptidoglycan layer, further restricting the penetration by AMPs [42,43]. This structural difference leads to its natural resistance to various AMPs, and often requires structural modification (e.g., fusion design or increasing hydrophobicity) to break through its outer membrane [43]. Therefore, the above-mentioned structural differences may be one reason why *Tj*Rcys1 was effective against Gram-positive bacteria, but only delayed the growth of Gram-negative bacteria and could not completely inhibit them.

Although the membrane components of *S*. *aureus* and *Bacillus* sp. T2 are different [44,45], *Tj*Rcys1 shows the same MIC value for these two Gram-positive bacteria. This might be because *Tj*Rcys1 acts on highly conserved and enriched negatively charged POPGs in the cell membrane [44,45]. Molecular simulation results indicate that the specific amino acid residues of *Tj*Rcys1 can form stable interactions with the phosphate groups of POPG (Figure 2), potentially disrupting the membrane structure and increasing membrane permeability. However, its specific mechanism of action still needs to be clarified through subsequent research.

The antibacterial activity detection in this study was conducted in 1 × PBS buffer. It is known that the activity of many AMPs is salt-dependent, and a high ionic strength environment may reduce their antibacterial efficacy by shielding the electrostatic interaction between peptides and bacterial membranes [46,47]. The net charge of *Tj*Rcys1 is +0.25, which is relatively low. This may further weaken its ability to bind to negatively charged bacterial membranes under high-salt conditions. Therefore, the moderate activity (MIC 64 μM) observed in PBS may be partly attributed to the high salt concentration under the detection conditions. Future studies evaluating the activity of *Tj*Rcys1 in low ionic strength buffers will help to more accurately assess its intrinsic antibacterial potential and clarify the mechanism by which salt concentration affects its activity. Consequently, the data currently identify *Tj*Rcys1 as a newly discovered member of the cysteine-rich AMP family, exhibiting detectable yet limited antibacterial activity, rather than demonstrating high potency suitable for immediate therapeutic use. Its primary significance lies in expanding the known structural diversity of AMPs derived from marine organisms and providing a foundational template for future mechanistic investigations and optimization efforts.

The number of positive charges and the hydrophobicity of AMPs are closely related to their antibacterial effects [48,49,50]. The net charge of *Pp*Rcys1 was +4.5, and GRAVY was 3.03. The net charge of *Tj*Rcys1 was +0.5, and GRAVY was 0.06. These data indicate that, when *Tj*Rcys1 interacts with the membrane, electrostatic attraction and a hydrophobic effect are weaker than those of *Pp*Rcys1, which might be one of the reasons for the relatively low antibacterial activity of *Tj*Rcys1. Experiments on membrane permeation and membrane mimic-binding indicated that the destructive effect of r*Tj*Rcys1 upon bacterial membranes was weaker than that elicited by r*Pp*Rcys1. Furthermore, MD simulations showed that at 100, 200, 300, 400, and 500 ns, the depth of *Tj*Rcys1 inserted into the membrane was less than that of *Pp*Rcys1, further supporting the conclusion made above. *Pp*Rcys1 contains 12 cysteine molecules, whereas *Tj*Rcys1 contains 11 cysteine molecules. Alphafold2 predicted that *Pp*Rcys1 could form six disulfide bonds [20], whereas the cysteine-pairing pattern of *Tj*Rcys1 is unclear and requires further research. MD simulations have indicated that the disulfide bond of *Pp*Rcys1 helps maintain structural stability during its insertion into a membrane [20]. All the cysteine molecules of *Tj*Rcys1 were located in the mature-peptide region and did not embed into the membrane during the 500 ns simulation. Therefore, although *Tj*Rcys1 contains multiple cysteine molecules and can form two β-folds, these cysteine molecules may not contribute directly to its antibacterial function.

A conserved structural motif known as the γ-core is widely observed in cysteine-rich AMPs [51]. This motif generally adopts a β-hairpin spatial conformation, stabilized by two disulfide-bonded pairs of conserved cysteine residues [52]. Based on sequence patterns, the γ-core can be classified into three subtypes: D-isomer, L1-isomer, and L2-isomer. Through sequence alignment and structural analysis, we identified an L2-isomer within residues 58–71 (CCCGEAFLGSKFCCKV) of *Tj*Rcys1, with the consensus sequence NH_2_…[C]–[X_3–9_]–[GXC]–[X_1–3_]…COOH [52]. This structural element is hypothesized to play an evolutionarily ancient and essential role in host–pathogen interactions [52]. The predicted γ-core region does not display distinct membrane-insertion behavior in the current snapshot of the molecular dynamics simulation, which may be attributed to its initial positioning and conformational state [53]. Consequently, the observations from the present simulation may not fully represent the conformational dynamics of this γ-core over extended timescales or under alternative initial conditions. Thus, the functional contribution of this motif to antibacterial activity requires further experimental validation, such as site-directed mutagenesis of the conserved domain or chemical synthesis of the corresponding peptide fragment for functional comparison.

Studies analyzing the inhibitory mechanism of *Tj*Rcys1 on the growth rate of Gram-negative bacteria, conducted through transcriptome sequencing, have revealed that r*Tj*Rcys1 treatment can cause significant differences in gene expression in *K. pneumoniae*. Among these DEGs, the genes with upregulated expression are mainly enriched in energy–metabolism pathways (e.g., metabolism of starch and sucrose, and glycolysis/gluconeogenesis), suggesting that bacteria may respond to environmental stress caused by peptide substances by activating the energy supply [54,55]. Genes with downregulated expression are mainly related to ribosomal biogenesis, nucleotide metabolism, and glycerophospholipid metabolism, indicating that r*Tj*Rcys1 may interfere with bacterial protein synthesis, metabolism of genetic material, and the stability of the cell-membrane structure [56,57,58]. Under the tested conditions where bacterial growth was not inhibited, these transcriptional alterations may reasonably reflect a general stress response rather than direct intracellular targeting by the peptide [59]. However, whether this is due to direct intracellular targeting or a secondary stress response remains to be elucidated. Such “metabolic reprogramming” is consistent with a pattern in which some AMPs affect bacterial physiological activities through multiple targets [32]. For instance, PR-39 can kill bacteria in a non-lytic manner by inhibiting the synthesis of proteins and DNA [60,61]. The human AMPs tPMP-1 and aHNP-1 can inhibit the synthesis of DNA and protein after entering cells [62,63]. In general, AMPs must enter bacterial cells to interfere with bacterial protein synthesis and the metabolism of genetic species [61,62]. Certain AMPs initially accumulate on the membrane surface and interact with lipid components. This interaction induces transient disruption of the membrane integrity, leading to dissipation of the transmembrane potential and the formation of a temporary toroidal pore [64]. Consequently, AMPs are internalized into the cytoplasm and ultimately reach their intracellular targets. Furthermore, according to the lipid phase boundary defect model, AMPs form planar aggregates on the surface of negatively charged bacterial membranes [65]. The insertion of aromatic residues into the hydrophobic core of the membrane promotes the generation of rigid lipid domains by these peptide aggregates. Discrepancies in mechanical properties, such as rigidity and thickness, between these domains and the surrounding membrane create structural defects, facilitating the translocation of AMPs across the bacterial membrane and into the cell [65,66]. However, the current transcriptomic findings do not conclusively demonstrate the intracellular entry of *Tj*Rcys1. Further experiments are required to distinguish between a direct intracellular mode of action and an indirect stress response mediated through membrane or extracellular interactions.

Recombinant *Tj*Rcys1 (r*Tj*Rcys1) expressed and purified in *E*. *coli* BL21(DE3) may not fully recapitulate the natural biosynthetic environment of this peptide. The correct pairing of the 11 cysteine residues within *Tj*Rcys1 is essential for its proper 3D structure and stability [16,67]. However, differences between the oxidative folding environment of *E*. *coli* and the endoplasmic reticulum environment in water fleas may affect the final spatial conformation and biological activity of r*Tj*Rcys1 [68]. Furthermore, natural peptides may undergo post-translational modifications such as glycosylation and phosphorylation, which can influence their stability and functional properties [68]. Prokaryotic expression systems generally lack the machinery required for such changes. Therefore, the observed activity of r*Tj*Rcys1 should be interpreted within the context of the specific expression and purification system employed. Future recombinant production using eukaryotic expression systems may enable the generation of peptides with conformations closer to the native form, thereby facilitating more accurate functional characterization.

Although molecular dynamics simulations and certain in vitro experiments have demonstrated that *Tj*Rys1 can interact with bacterial cell membranes, and transcriptomic analyses suggest potential intracellular targets, the precise mechanisms underlying its interaction with membrane lipids or intracellular molecules, as well as its specific molecular targets, remain to be fully elucidated. Current research has primarily focused on characterizing the in vitro antibacterial activity and initial mechanistic insights, yet a comprehensive evaluation of its in vivo efficacy, toxicity profile, metabolic stability, and activity against multidrug-resistant bacterial strains remains lacking. These aspects will constitute critical directions for future investigations. Future research may consider enhancing the membrane-targeting activity of *Tj*Rcys1 through site-directed mutagenesis or sequence optimization, such as introducing positively charged residues or adjusting hydrophobicity [69,70]. One could explore the synergistic effect of *Tj*Rcys1 with other antibacterial agents [71], or utilize nano-delivery systems to enhance its local concentration and targeting. Meanwhile, it is necessary to clarify further its intracellular action targets and the regulatory mechanisms of signaling pathways.

The assessment of the antibacterial activity of *Tj*Rcys1 in this study was limited to several common Gram-positive and Gram-negative bacteria. Although these tested strains are representative, their scope is relatively limited, and they fail to cover other critical pathogenic microorganisms in aquaculture or clinical Settings. Therefore, the breadth of the antibacterial spectrum of *Tj*Rcys1 and its specificity for different pathogens still need to be verified in a broader range of strains. Future studies should include a wider range of pathogenic organisms to enable a more comprehensive assessment of the biological potential of *Tj*Rcys1.

## 4. Materials and Methods

### 4.1. Bacterial Strains and Culture Conditions

The bacterial strains used in this study comprised two Gram-positive species—*S. aureus* (ATCC 6538) and *Bacillus* species T2—and four Gram-negative strains: *A. hydrophila* (ATCC 35654), *V. alginolyticus* (ATCC 17749), *K. pneumoniae* (CICC 10493), and *E. coli* (ATCC 8739). All strains except *K. pneumoniae* were provided by Professor Chaogang Wang [72]. *K. pneumoniae* was sourced from the China Industrial Microbial Culture Collection and Management Center (Beijing, China).

All strains were maintained as glycerol stocks at −80 °C to ensure long-term viability and stability. Before use, each strain was revived by inoculating 50 μL of frozen stock into 2 mL of appropriate liquid medium and incubating at 37 °C with shaking at 200 rpm for 12 h to achieve active logarithmic growth. Routine cultivation was carried out in Luria–Bertani (LB) broth (ST163; Beyotime, Shanghai, China) for *S. aureus*, *Bacillus* species T2, *E. coli*, *K. pneumoniae*, and *A. hydrophila*, whereas *V. alginolyticus* was cultured in Zobell Marine Agar 2216E medium (HB0132; Haibo, Qingdao, China).

### 4.2. Prediction and Identification of Cysteine-Rich AMPs

The genome data of *T. japonicus* were retrieved from the National Center for Biotechnology Information (NCBI) database (accession number: GCA_010645155.1). In general, cysteine-rich AMPs are composed of ~100 amino acids and are localized in the extracellular environment [67]. Peptides of this class, including Mersacidin, Laterosporulin, Subtilosin A, Thuricin CDα, Thuricin CDβ, and Thuricin H, demonstrate potent antibacterial effects and usually feature cysteine content > 10% [73].

In the present study, a custom-built computational pipeline was applied to screen candidate sequences based on three criteria: length < 100 amino acids, lack of functional annotation in the NCBI BLAST database, and cysteine proportion > 10%. Signal peptides were predicted using SignalP 6.0. Subsequently, candidate sequences were ranked based on their net charge, and the top-scoring sequence was designated “*Tj*Rcys1”. The putative antibacterial activity of *Tj*Rcys1 was assessed using the “prediction” function in the APD3 database. Structural modeling of *Tj*Rcys1 was done with AlphaFold2. In addition, key physicochemical properties of the peptide were analyzed using ProtParam (https://web.expasy.org/protparam/, accessed on 1 October 2025) and the APD3 Database.

### 4.3. MD Simulations

A model of a cell membrane was constructed using CHARMM-GUI [74]. A mixed membrane was generated at a ratio of POPE: POPG = 3:1. Subsequently, two simulation boxes with dimensions of 9.074 × 9.452 × 7.716 nm^3^ and 11.882 × 11.882 × 15.530 nm^3^ were constructed using Gromacs 2023.3 [75]. We placed a *Tj*Rcys1 molecule at the center of the first box. In the second box, we positioned the mixed cell membrane at the center and put one *Tj*Rcys1 molecule 2 nm above the membrane surface. Subsequently, water molecules were added to the system for solvation, and an appropriate amount of Na+ and Cl− was added to neutralize the net charge of the system. Simultaneously, the salt concentration was adjusted to the physiological concentration of 0.15 mol/L. *Tj*Rcys1, POPE, and POPG molecules were described using the CHARMM36 force field [76]. The water molecule was described using the TIP3P water model [77]. The topological parameters of *Tj*Rcys1, POPE, and POPG were generated through the pdb2gmx program within Gromacs 2023.3 [75].

All MD simulations were completed in Gromacs 2023.3 [75]. First, the system was subjected to energy minimization (using the steepest descent method with a maximum force tolerance of 1000 kJ/mol/nm). Then, pre-equilibrium simulations of 500 ps were conducted successively under NVT and NPT ensembles. After pre-balancing, a formal simulation of 200 ns was conducted for the system in a single aqueous solution. In contrast, a formal simulation of 500 ns was carried out for the membrane–peptide composite system. The simulated temperature was set at 310.15 K (37 °C), and the V-rescale [78] temperature controller was used for temperature coupling. The pressure was controlled at 1 bar by the C-Rescale [79] pressure controller. The cutoff distance of van der Waals interactions was set at 12 Å. Electrostatic interactions were handled using the particle mesh Ewald method [80,81]. The simulation step size was 2 fs. The trajectory was saved every 10 ps. Trajectory “snapshots” were extracted and analyzed through VMD [82]. All simulations applied periodic boundary conditions in the x, y, and z directions.

### 4.4. Expression and Purification of Recombinant TjRcys1 (rTjRcys1)

DNA sequences encoding the *Tj*Rcys1 (GenBank accession number: PX684235) were codon-optimized for expression in *E. coli* and flanked with BamHI and XhoI restriction sites at the 5′ and 3′ ends. These sequences were synthesized by General Biosystems (Chuzhou, China) and cloned into the pSmartI vector, which carries a His-SUMO fusion tag, using digestion and ligation with BamHI and XhoI. The resulting plasmid, named “pSmartI-*Tj*Rcys1” (5847 bp in length; Supplementary Figure S1), was transformed into *E. coli* BL21 (DE3) cells via heat-shock transformation. Transformed colonies were selected on LB agar plates containing kanamycin (50 µg/mL). Positive clones were verified by polymerase chain reaction (PCR) amplification and DNA sequencing before being used for the production of recombinant proteins. Detailed primer designs and PCR conditions are listed in Supplementary Tables S2 and S3. Protein expression was induced by adding IPTG to a final concentration of 0.5 mM, followed by incubation for 12 h at 16 °C.

Cells were harvested and lysed using TieChui *E. coli* Lysis Buffer (ACE Biotechnology, Changzhou, China). They were then centrifuged (8000× *g*, 30 min, 4 °C) to separate soluble proteins (supernatant) from cell debris (pellet). Crude extracts from non-induced and induced cultures were analyzed by SDS–PAGE using 12% gels to confirm expression. The soluble His-SUMO-*Tj*Rcys1 fusion protein in the supernatant was purified using nickel-affinity chromatography. The eluted fractions were dialyzed against 1× phosphate-buffered saline (PBS) for 18 h at 4 °C. To cleave the His-SUMO tag, 1 unit of SUMO protease (General Biosystems, Chuzhou, China) was added, and the solution was incubated for 6 h at 4 °C. During subsequent passage through the nickel column, the cleaved His-SUMO tag was retained, whereas the flow-through fraction containing the tag-free recombinant r*Tj*Rcys1 was collected. The purified protein was analyzed by SDS–PAGE, and its concentration was determined using a bovine serum albumin (BSA)-based protein assay kit (Beyotime) according to the manufacturer’s protocols. Finally, the purified r*Tj*Rcys1 was lyophilized and stored at −80 °C until further use. The freeze-dried polypeptide powder was entrusted to Wininnovate Bio Company (Shenzhen, Guangzhou, China) for qualitative identification by LC-MS.

### 4.5. Antimicrobial Activity Assay of Recombinant TjRcys1

Only the recombinant *Tj*Rcys1 was tested, and its natural form was not involved. To evaluate the minimum inhibitory concentration (MIC) of r*T*jRcys1, a modified microtiter plate method was carried out following the guidelines of the Clinical and Laboratory Standards Institute (Beijing, China) [83]. Bacterial cultures were grown to an OD_600_ of 0.4 and subsequently adjusted to a final concentration of 10^4^ CFU/mL in Mueller–Hinton broth (HB6232; Haibo, Qingdao, China). The recombinant peptide r*Tj*Rcys1 and its variants were dissolved in 1× PBS. In each well of a 96-well plate, 20 μL of the peptide solution was mixed with 80 μL of the diluted bacterial inoculum. A twofold serial dilution series (64, 32, 16, 8, 4, 2, 1 μM) was applied to assess antimicrobial activity. Ampicillin served as the positive control. 1× PBS was used as the negative control. The plates were incubated for 18 h at 37 °C. The MIC was determined based on the lack of microbial growth, as detected by resazurin staining and measured by optical density at OD_560_ and OD_590_. Experiments were conducted with three biological replicates and three technical replicates per condition. After the MIC experiment, the OD_600_ values of the four test Gram-negative bacteria at 64 μM were determined at 0, 4, 8, 12, 24, and 48 h, respectively, which were used to construct the bacterial growth curve. r*Pp*Rcys1 at 64 μM was used as the positive control. BSA at 64 μM was used as the negative control. Experiments were subjected to three biological and technical replicates.

### 4.6. Assay to Measure the Binding of Mimetics to Membranes

In accordance with our previous method [84], we wished to optimize the lipid composition. Hence, a molar ratio of 3:1 nmol of POPE to POPG was selected, resulting in a total lipid concentration of 100 μM. Initially, lipids were dissolved in chloroform, followed by solvent removal under a gentle stream of nitrogen. The formed lipid film was further desiccated under high vacuum for 1 h to ensure the complete elimination of residual organic solvents. Hydration of the dried lipid film was undertaken using preheated HEPES buffer (HEPES (20 mM), NaCl (150 mM), pH 7.4). The mixture was vigorously vortex-mixed and sonicated to produce small, uni-lamellar vesicles. Sonication was performed using either probe sonication with 10 cycles of 10 s pulses on ice or bath sonication at 55 °C for 30 min. Then, the liposomal suspension was diluted to a working concentration of 10–20 μg/mL and dispensed into a 96-well microplate at 100 μL per well. Plates were incubated overnight at 4 °C to facilitate the adsorption of liposomes onto the well surfaces. After adsorption, wells were washed thrice with 1× PBS containing 0.05% Tween 20 (PBST; pH 7.4; 60146ES76; Yeasen, Shanghai, China). To reduce nonspecific interactions, each well was blocked with 100 μL of 5% skim milk in 1× PBST (pH 7.4) and incubated for 2 h at 37 °C. Following the blockade, wells were washed gently three times with 1× PBST to remove excess blocking solution prior to the assay.

Direct detection of binding activity was not feasible due to the absence of affinity tags in r*Tj*Rcys1. Therefore, His-SUMO-*Tj*Rcys1 fusion protein was used for binding assessment. The His-SUMO moiety served as a control. His-SUMO-*Tj*Rcys1 was diluted to 10 μM in 1× PBS (pH 7.4) and added to designated wells. As a positive control, His-SUMO-*Pp*Rcys1 (10 μM) was applied, while His-SUMO tag (10 μM) alone served as the negative control. The plate was incubated for 1 h at 37 °C, followed by a single wash with 1× PBST (pH 7.4). Next, 100 μL of horseradish peroxidase-conjugated anti-His antibody, diluted 1:5000 in 1× PBST (pH 7.4; Boyi, Changzhou, China), was added to each well. After incubation for 1 h at 37 °C, the unbound antibody was removed by five washes with 1× PBST. For signal development, 100 μL of 3,3′,5,5′-Tetramethylbenzidine (TMB) substrate solution was added to each well to initiate color formation. The reaction was stopped by the addition of 200 μL of ELISA stop solution per well. Optical density was measured at 450 nm using a microplate reader (Synergy™ LX; BioTek, Kaysville, UT, USA).

### 4.7. Assay to Measure Membrane Permeability

Globally, *S*. *aureus* is a primary pathogen responsible for bacterial foodborne intoxication and nosocomial infections [45]. The increasing prevalence of antimicrobial resistance in this organism further complicates treatment strategies, underscoring the urgency of research into effective prevention and control measures [45]. *Bacillus* sp. T2 is an environmental isolate exhibiting lower pathogenic potential compared to *S*. *aureus* [85]. Therefore, the present study focused exclusively on assessing the effects of r*Tj*Rcys1 on membrane permeability in *S*. *aureus*, complemented by scanning SEM analysis. The membrane-disrupting effect of r*Tj*Rcys1 upon *S. aureus* was evaluated using an assay to measure LDH release. Bacterial cells in mid-logarithmic growth phase (OD_600_ ≈ 0.5) were harvested, washed, and resuspended in PBS. A 100-μL aliquot of the bacterial suspension was transferred to a 96-well plate and treated with r*Tj*Rcys1 (64 μM) for 2 h. Following incubation, samples were centrifuged (12,000× *g*, 2 min, 30 °C). Then, 50 μL of the supernatant was mixed with an equal volume of reaction solution containing sodium phosphate buffer (50 mM, pH 7.5), pyruvate (0.6 mM), and Nicotinamide adenine dinucleotide (0.2 mM). The mixture was incubated for 10 min at room temperature, after which the reaction was stopped by the addition of 50 μL of acetic acid (1 M). Optical density was measured at 340 nm using a microplate reader (Spark; Tecan; Mannedorf, Switzerland).

Complete lysis of bacterial cells induced by 1% Triton X-100 served as the reference for total LDH release [86]. r*Pp*Rcys1 (64 μM) was used as the positive control. BSA was the negative control. The extent of membrane disruption elicited by r*Tj*R*cys1* was expressed as the percentage of LDH released, calculated as the ratio of LDH activity in treated samples to that in fully lysed cells, using the following formula:

$$\mathrm{Permeability}\;(\%)\;=\;\frac{OD340\left(Sample\right)-OD340(BSA)}{OD340\left(Triton\;X-100\;treated\right)-OD340(BSA)}\times 100\%$$

Controls were bacteria treated by BSA (negative) and 1% Triton X-100 (positive).

### 4.8. Propidium Iodide (PI) Staining

*S. aureus* was grown as previously described to its MIC and incubated for 4 h. The samples were then labeled using a PI staining kit (Sangon, Shanghai, China) according to the manufacturer’s protocol. Fluorescence microscopy was performed to visualize the cells using a BX51 microscope (Olympus, Tokyo, Japan).

### 4.9. SEM

SEM was conducted according to protocols [84]. *S. aureus* cultures were grown in LB broth until they reached the mid-log phase of growth. Then, cells were collected by centrifugation (5000 rpm, 3 min) and resuspended in 1× PBS to obtain a final density of 10^6^ CFU/mL. Aliquots of the bacterial suspension were incubated with r*Tj*Rcys1 (64 μM) for 2 h on circular coverslips placed in 24-well plates. After incubation, samples were fixed overnight at 4 °C with 5% glutaraldehyde prepared in PBS (pH 7.4), followed by three washes with 1× PBS. As a negative control, bacteria treated with BSA under identical conditions were included. For dehydration, an ascending ethanol series (30%, 50%, 70%, 80%, 90%, 100%) was applied at 4 °C, with each step maintained for 10 min. Subsequently, specimens were dried using a critical point dryer (HCP; Hitachi, Tokyo, Japan), sputter-coated with a fine gold layer (MC1000; Hitachi), and visualized using a scanning electron microscope (APREO S; Thermo Fisher Scientific, Waltham, MA, USA).

### 4.10. Total RNA Extraction, Illumina Sequencing, and Analyses

*K. pneumoniae* was cultured in LB broth to an OD_600_ of 0.4, and then diluted with fresh Mueller–Hinton broth to adjust the bacterial solution concentration to 1 × 10^4^ CFU/mL. The control group was treated with BSA (64 μM). The experimental group was treated with recombinant protein r*Tj*Rcys1 (64 μM). The treatment time for both groups was 24 h. Three independent biological replicates were set for each group. After processing, we collected the bacterial cells, centrifuged them (5000 rpm, 3 min), and rapidly froze them in liquid nitrogen. Total RNA was extracted using the RNeasy Mini Kit (Qiagen, Hilden, Germany). Genomic DNA was removed by DNase (Qiagen) treatment. RNA integrity was strictly controlled by a bioanalyzer (2100 series; Agilent Technologies, Santa Clara, CA, USA). After the qualified total RNA was enriched by removing rRNA, the fragmentation buffer was added to randomly break the mRNA into shorter fragments.

After library construction was complete, a preliminary quantification was done using a fluorometer (Qubit 2.0; Thermo Fisher Scientific). Samples were diluted to 1.5 ng/μL, and then the size of the inserted fragments was detected using a bioanalyzer (2100 series; Agilent Technologies). After meeting expectations, the effective concentration of the library (greater than 1.5 nM) was accurately quantified using real-time reverse transcription-quantitative polymerase chain reaction. After library inspection, different libraries were mixed according to the effective concentration and the required sequencing data volume for Illumina sequencing. Raw reads were processed by the fastp software (v1.0.0) to remove reads with linkers [87], ploy-N, and low quality: clean reads were obtained. We downloaded the reference genome and annotation files of *K. pneumoniae* from NCBI (https://ftp.ncbi.nlm.nih.gov/genomes/all/GCF/022/869/665/GCF_022869665.1_ASM2286966v1/, accessed on 15 September 2025), and aligned the clean reads to the reference genome using Bowtie2 (2.5.4) [88]. We calculated the readings mapped to each gene through featureCounts (2.0.6) [89]. Then, we calculated the FPKM based on the gene length to evaluate gene expression. Inter-group differential expression was conducted using DESeq2 (1.42.0), with p_adj_ ≤ 0.05 and log_2_ (fold change) ≥ 0 as the threshold [90]. Finally, clusterProfiler (4.4.2) was used to conduct analyses of signaling-pathway enrichment based on the Kyoto Encyclopedia of Genes and Genomes (www.genome.jp/kegg/pathway.html, accessed on 14 November 2025) on DEGs [91].

### 4.11. Statistical Analyses

Data were analyzed using Prism 10.0 (GraphPad, San Diego, CA, USA). Significance was assessed through one-way analysis of variance. Data are the mean ± standard deviation. *p* < 0.05 was considered significant.

## 5. Conclusions

Based on the genomic data of *T. japonicus*, we screened and characterized a cysteine-rich AMP: *Tj*Rcys1. This AMP was composed of 96 amino acids. Prediction of three-dimensional structure showed that it contained mainly random curls and had two α-helices and two β-folds, and the overall surface was negatively charged. The recombinant expression of *Tj*Rcys1 showed inhibitory effects upon *S. aureus* and *Bacillus* species T2, and the MIC was 64 μM, respectively. At 64 μM, *Tj*Rcys1 did not completely inhibit *V. alginolyticus*, *K. pneumoniae*, or *A. hydrophila*, but it could slow down their growth rates. *Tj*Rcys1 could disrupt the integrity of the cell membrane of *S. aureus*. Transcriptomic analyses revealed that *Tj*Rcys1 could affect the ribosomal biosynthesis pathway and nucleotide metabolism of *K. pneumoniae*. Our results identify *Tj*Rcys1 as a newly discovered cysteine-rich AMP with measurable but limited antimicrobial potency, providing foundational data for understanding the diversity of AMPs in marine crustaceans and for future rational design efforts.

## Figures and Tables

**Table 1:** Minimal inhibitory concentration (MIC) of r*Tj*Rcys1 against Gram-positive and Gram-negative bacteria.

Microorganism		MIC (μM)	
		r*Tj*Rcys1	r*Pp*Rcys1
Gram-positive bacteria	*S. aureus*	64	8
	*Bacillus* sp. T2	64	8
Gram-negative bacteria	*A. hydrophila*	–	32
	*K. pneumoniae*	–	64
	*E. coli*	–	16
	*V. alginolyticus*	–	16

At a concentration of 64 μM, the MIC could not be determined, as indicated by “–” in the table. Only the reconstituted expression of *Tj*Rcys1 was tested, and its natural form was not involved.

## Data Availability

All data generated or analyzed during this study are included in this published article/Supplementary Material, and further inquiries can be directed to the corresponding authors.

## Supplementary Materials

The following supporting information can be downloaded at: https://www.mdpi.com/article/10.3390/md24010045/s1, Figure S1: pSmartI-*Tj*Rcys1_RMRK map; Table S1: Result of Screening of AMPs with High Cysteine Content; Table S2: General Primers of pSmartI; Table S3: PCR amplification program; Table S4: Differentially expressed genes of *K. pneumoniae* in the jRcys1 treatment group and the control group.
