# Supplementary material for: Recombinant Expression and Antimicrobial Mechanism of Cysteine-Rich Antimicrobial Peptides from Tigriopus japonicus Genome
Source: Mar Drugs. 2026 Jan 16;24(1):45. doi: 10.3390/md24010045 (PMC12842719; doi:10.3390/md24010045)
Supplement: Supplementary file 1 [file marinedrugs-24-00045-s001.zip › supplementary Table S1.pdf]

Supplementary Table S1. Result of Screening of AMPs with High Cysteine Content

Name	ID in Genomes files	Sequences	APD3 prediction results	Net charge
*Tj*Rcys1	Tj_106650T	MKIVFGVFVILSIFTIGTWATDPEIVEDRQSFIGGGELFSSYFNDCTRQFCPTY<br>KHRCCCEGAFLGSKFCCKVRTNSQVCSLYTCRDGYWQCAQDC	YES	+0.25
*Tj*Rcys2	Tj_054560T	QGGCSLVSDATMKGTCMTASECSSKGGSADGNCASGFGVCCSFVIKGCGG<br>TISHNCTYVQNTEFPASTEGGQTCKFGFNRICDSEYMIQQ	YES	-1.75
*Tj*Rcys3	Tj_040070T	MVLVGMVWWTHPEVQGQPTQFPECQCFAACPVGTCSNWTLEGQCCDNL<br>AECSPGGCCDDICQVDADCKMGPCGTCVKQEPEDQEGRCGRSRLANEQ	NO	-8.75
*Tj*Rcys4	Tj_094910T	MKSCAVWILFLSLDLIEANIRCYSCAPCNEFEHFAGDLSYFERDCFLDRSCM<br>KITGTTRDEYGYEREVSVRGCPVISGLTNLEQGCTETSFQCEHTSCF	NO	-6.5
