# Supplementary material for: Recombinant Expression and Antimicrobial Mechanism of Cysteine-Rich Antimicrobial Peptides from Tigriopus japonicus Genome
Source: Mar Drugs. 2026 Jan 16;24(1):45. doi: 10.3390/md24010045 (PMC12842719; doi:10.3390/md24010045)
Supplement: Supplementary file 1 [file marinedrugs-24-00045-s001.zip › supplementary Table S2.pdf]

Supplementary Table S2. General Primers of pSmartI

Primers	EF	ER
EF	TTA AGA TTC TTG TAC GAC GG	TGC TAG TTA TTG CTC AGC GG
