# Supplementary material for: Recombinant Expression and Antimicrobial Mechanism of Cysteine-Rich Antimicrobial Peptides from Tigriopus japonicus Genome
Source: Mar Drugs. 2026 Jan 16;24(1):45. doi: 10.3390/md24010045 (PMC12842719; doi:10.3390/md24010045)
Supplement: Supplementary file 1 [file marinedrugs-24-00045-s001.zip › supplementary Table S3.pdf]

Supplementary Table S3. PCR amplification program

| Program | Temperature | Time | Cycles |
|---|---|---|---|
| Initial denaturation | 95 ℃ | 5 min | 1 |
| Denaturation | 95 ℃ | 30 s | 35 |
| Anneal | 51 ℃ | 30 s | |
| Extend | 72 ℃ | 1 min | |
| Final extension | 72 ℃ | 5 min | 1 |
